# Brain Microvascular Endothelial Cell-Derived Exosomes Protect Neurons from Ischemia–Reperfusion Injury in Mice

**DOI:** 10.3390/ph15101287

**Published:** 2022-10-19

**Authors:** Jin Sun, Qing Yuan, Lichen Guo, Guangxu Xiao, Tong Zhang, Bing Liang, Rongmei Yao, Yan Zhu, Yue Li, Limin Hu

**Affiliations:** State Key Laboratory of Component-Based Chinese Medicine, Ministry of Education Key Laboratory of Pharmacology of Traditional Chinese Medicine Formulae, Institute of Traditional Chinese Medicine, Tianjin University of Traditional Chinese Medicine, Tianjin 301617, China

**Keywords:** brain endothelial cells, exosomes, ischemic stroke, synaptic plasticity, apoptosis

## Abstract

Stroke often results in neurological and neuropsychiatric sequela. Exosomes derived from brain endothelial cells (EC-Exo) protect neurons from hypoxic injury. However, the biological role of exosomes in apoptosis and synaptic plasticity remains unclear. This research aimed to assess whether cerebral microvascular endothelial cells inhibit apoptosis and promote synaptic remodeling through exosome-mediated cell–cell interaction after the ischemic attack. The effects of EC-Exo on primary neuronal apoptosis and synapses in oxyglucose deprivation reoxygenation (OGD/R) injury were first assessed in vitro. Animal experiments were performed using C57BL/6J mice, divided into three groups: a sham group, a model (middle cerebral artery occlusion/reperfusion, MCAO/R) group, and an EC-Exo group (tail vein injection of EC-Exo, once/2 days for 14 days) to evaluate the neuromotor and exploratory abilities of mice after MCAO/R. Apoptosis and synaptic protein expression levels were detected. The results demonstrated that EC-Exo inhibited neuronal apoptosis and increased synaptic length after OGD/R. In vivo, EC-Exo not only improved neural motor behavior and increased regional cerebral blood flow (rCBF) in MCAO/R-injured mice but also promoted the expression of synaptic regulatory proteins and inhibited apoptosis in the brain. These results suggest that EC-Exo may provide neuroprotection against stroke by promoting synaptic remodeling and inhibiting apoptosis from protecting neurons.

## 1. Introduction

Stroke remains one of the leading causes of death and disability worldwide [1]. Ischemic strokes (IS) account for approximately 87% of all strokes [2] and often result in neurological and neuropsychiatric sequelae [3,4]. In addition, cognitive impairment is prevalent after stroke, and clinical studies have displayed that cognitive decline is faster in stroke patients than in non-stroke patients within 1–3 years of stroke onset [5].

The treatment of acute ischemic stroke (AIS) has changed dramatically with the development of intravenous alteplase thrombolysis and endovascular thrombolysis [6,7]. These treatments open occluded vessels, and blood perfusion to the ischemic tissue is restored. An alternative option for treating AIS is to target the ischemic cascade directly. In the past, this approach was known as neuroprotection. However, it may be more appropriate to refer to it as cytoprotection [8]. All cells in the area of ischemia, including neurons, endothelial cells, pericytes, astrocytes, and microglia, are at risk of injury [9]. The neurons are likely to be the most susceptible of these cells, and their death contributes significantly to the clinical deficits associated with AIS [10].

Intercellular communication and signaling in the brain are fundamental to the central nervous system (CNS) homeostasis and function. Endothelial cells are distributed throughout the vascular network. It is a “first responder” to hypoxic stress [11] and is highly involved in sending paracrine signals to body tissues. However, due to the complexity of the neurovascular interactions, understanding post-stroke regulation is still in its early stages.

Exosomes are small cellular membrane vesicles secreted by cells, between 30–200 nm in diameter, capable of crossing the blood–brain barrier (BBB) [12]. Exosomes carry proteins, nucleic acids, and lipids. Under physiological and pathophysiological conditions, they play a crucial role in intercellular communication by transporting these cargoes between source and target cells [13]. Vascular endothelial cells are one of the major cell types that release extracellular vesicles into the bloodstream [14]. However, their physiological significance is still unknown.

Previous studies have revealed that exosomes from human umbilical vein endothelial cells contain Dll4 protein, which stimulates Notch3 receptors on pericytes, protects the structural stability of cerebral blood vessels, and regulates vascular regeneration [15]. Under ischemia/reperfusion (I/R) conditions, exosomes released from femoral artery endothelial cells promoted Bcl-2 expression, inhibited Bax and Caspase-3 expression in neuronal cells SH-SY5Y, and promoted cell proliferation, migration, and invasion, thus protecting neuronal cells from I/R damage [16]. In addition, EC-Exo markedly improved cognitive and neurological function in a type 2 diabetic mouse model, increasing arterial diameter, vascular density, and axonal density [17]. However, the biological role of exosomes in apoptosis and synaptic plasticity remains unclear.

This study established an animal model of mouse MCAO/R by wire-plug method and an OGD/R cell model of primary neuronal cells. In addition, the role of EC-Exo in apoptosis and synaptic remodeling after in vivo and in vitro hypoxic injury was assessed. These findings contribute to understanding the mechanism of action of EC-Exo against I/R damage.

## 2. Results

### 2.1. Isolation and Identification of Exosomes

**Figure 1:**
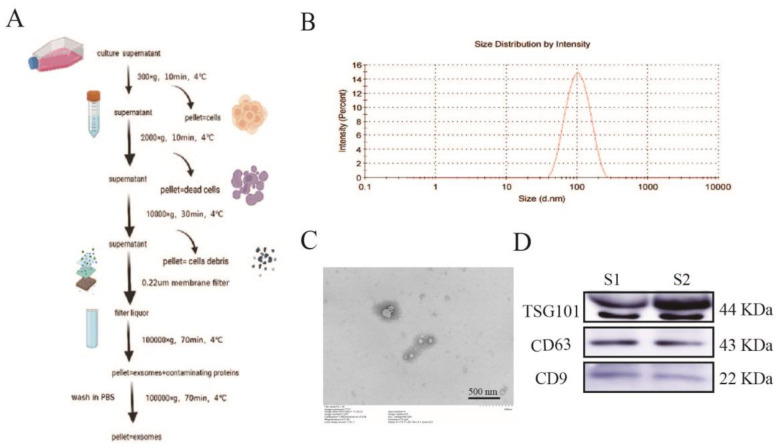
Extracting and identifying the exosomes. (**A**) Brain microvascular endothelial cell exosome extraction and purification process. (**B**) Detecting the nanodiameter range and average size of exosomes by DLS. (**C**) Microscopic morphology of bEnd.3 observed by transmission electron microscopy. Scale bar = 500 nm. (**D**) Immunoblotting revealed that isolated exosomes expressed TSG101, CD9, and CD63. n = 2. DLS: dynamic light-scattering particle size analyzer.

Consistent and highly homogeneous exosomes were obtained by differential centrifugation (Figure 1A) and characterized by transmission electron microscopy (TEM), dynamic light-scattering particle size analyzer (DLS), and protein blot analysis. The DLS results revealed that the average particle size of exosomes was 89 nm (Figure 1B). Exosomes under TEM demonstrated small membrane vesicles ranging from 30 to 150 nm in diameter (Figure 1C). Western blot analysis demonstrated that the exosomes expressed CD9, CD63, and TSG101 (Figure 1D).

### 2.2. Identification of Primary Neurons and EC-Exo Protects Neurons from OGD/R Damage

**Figure 2:**
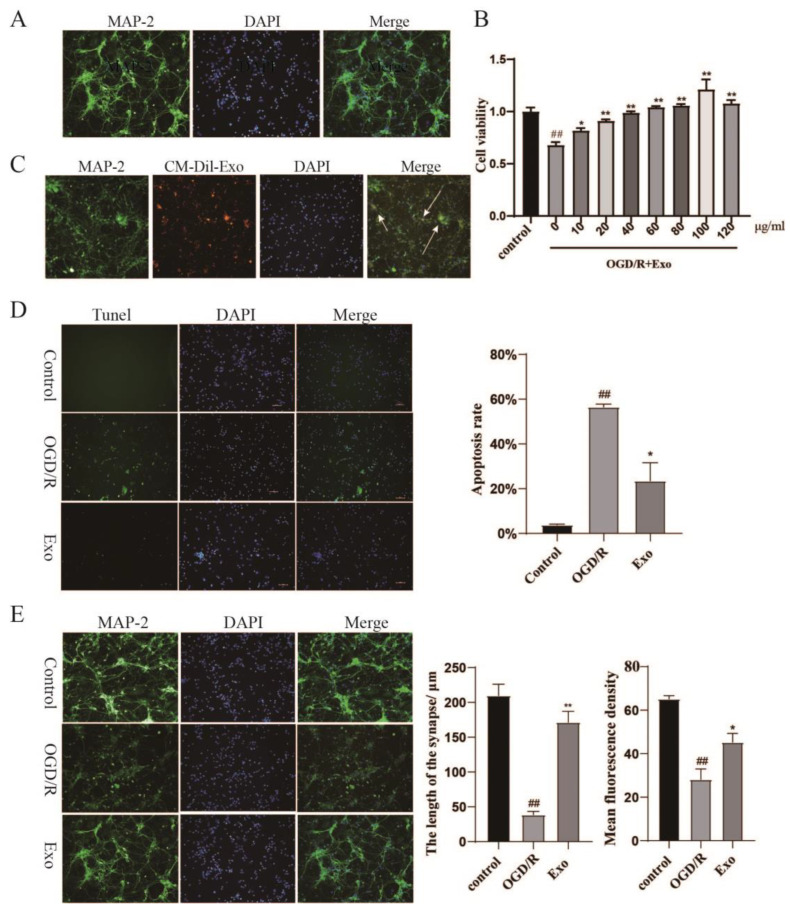
EC-Exo protects mouse primary neuronal cells from ischemic injury. (**A**) Identifying the purity of MAP2 primary neurons, the neuronal cells were morphologically normal and over 95% pure. Magnification ×200, Scale bar = 50 μm. (**B**) Primary neuronal OGD/R model establishment and the effect of exosomes on neuronal OGD/R injury cell viability. (**C**) Uptake of CM-Dil-labeled EC-Exo by MAP2-labeled neurons (green) (white arrowheads). Magnification ×200, scale bar = 50 μm. (**D**) TUNEL immunofluorescence assay to detect apoptosis and apoptotic number statistics of primary neurons. Magnification ×200, scale bar = 50 μm. (**E**) Statistical results of synaptic length and fluorescence density after OGD/R injury of primary neurons. Magnification ×200, Scale bar = 50 μm. All data are expressed as mean ± marked difference (n = 3). The data were analyzed by one-way ANOVA followed by multiple comparisons using Tukey’s post hoc test. ## *p* < 0.01 vs. sham; * *p* < 0.05, ** *p* < 0.01 vs. model. EC-Exo: Exosomes derived from brain endothelial cells; OGD/R: oxyglucose deprivation reoxygenation.

After 4 h of inoculation, most cells began to adhere to the well, and a few developed short protrusions. After 7–8 days, synaptic branches increased, and closer connections were established between cells. The cells were identified using MAP-2 immunofluorescence chemistry, and the results demonstrated that the neuronal cells were morphologically normal and over 95% pure (Figure 2A). To investigate the role of EC-Exo in neuroprotection, we treated neuronal cells after OGD/R injury with EC-Exo for 24 h. CCK-8 demonstrated a cell survival rate of 71.53% after 2 h of OGD injury. EC-Exo treatment significantly increased the cell survival rate. The most significant effect was observed at a concentration of 100 μg/mL (*p* < 0.05). The concentration was chosen for subsequent experiments (Figure 2B).

### 2.3. EC-Exo Increases the Neuronal Synaptic Length and Inhibits Apoptosis In Vitro

We investigated whether primary mouse neurons could take up EC-Exo. CM-Dil-labeled exosomes were incubated with neurons for 12 h. Fluorescence microscopy demonstrated that CM-Dil-labeled exosomes (orange) were localized in the cytoplasm of neuronal cells (green), indicating that the exosomes could be taken up by neurons (Figure 2C).

TUNEL staining revealed a decrease in apoptosis in neurons after EC-Exo treatment compared to the model group (*p* < 0.05) (Figure 2D). Second, immunofluorescence staining demonstrated an increase in synaptic length of neurons after EC-Exo treatment compared to the model group (*p* < 0.05) (Figure 2E).

### 2.4. EC-Exo Treatment Improves Pathology in MCAO/R Mice

**Figure 3:**
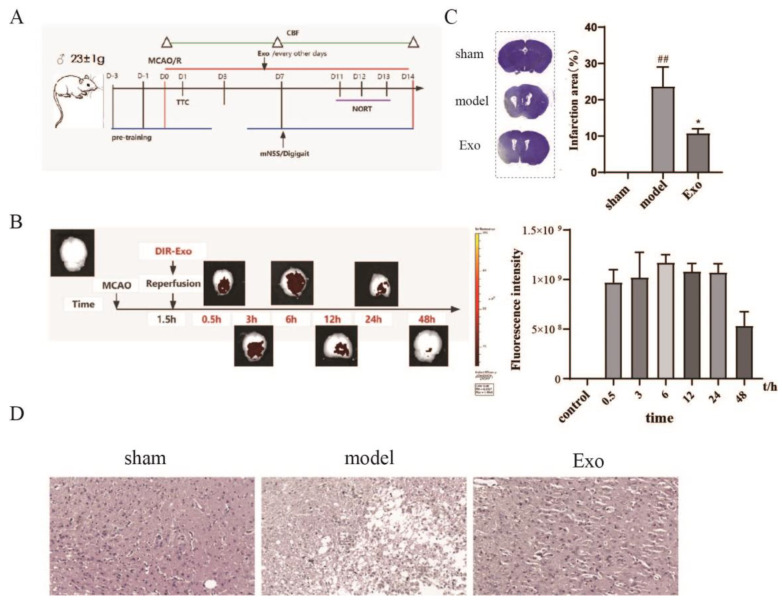
EC-Exo reduces brain infarct volume and improves pathological morphology in MCAO/R mice. (**A**) Animal experimental design. (**B**) Results of DIR fluorescence-labeled exosome intracerebral tracing experiments. (**C**) Crystal violet staining of representative brain sections (infarct areas are lighter in color) at 14 days after MCAO/R. (**D**). Histological examination of HE staining 14 days after MCAO/R, magnification ×200, scale bar = 20 μm. All data are expressed as mean ± marked difference (n = 4). The data were analyzed by one-way ANOVA followed by multiple comparisons using Tukey’s post hoc test. ## *p* < 0.01 vs. sham; * *p* < 0.05 vs. model. EC-Exo: exosomes derived from brain endothelial cells; MCAO/R: middle cerebral artery occlusion/reperfusion.

The design of the animal experiment is displayed in Figure 3A. An MCAO/R model was established. We first observed whether the tail vein injection of EC-Exo could reach the brain of mice and the metabolism of exosomes in the brain at 48 h. DIR-labeled exosomes were already present in the brain 30 min after tail vein administration, and the total number of exosomes reaching the brain increased as the time was extended; the total number of exosomes decreased at 24 h, and a small number of exosomes remained at 48 h. The results indicate that exosomes can reach the brain of mice (Figure 3B). Thus, we determined the frequency of administration to be every 48 h.

We evaluated the effect of EC-Exo on brain infarct volume in MCAO/R mice by crystalline violet staining and found that EC-Exo could significantly reduce brain infarct volume. The brain infarct volume of mice in the EC-Exo group was significantly reduced compared with that of the model group (Figure 3C). In addition, we examined HE-stained brain tissue to assess the effect of EC-Exo and found that the path morphology was significantly improved after EC-Exo treatment. As demonstrated in Figure 3D, compared with the model group, the normal cells in the ischemic brain tissue of mice in the EC-Exo group were more numerous and more closely arranged, with clearer nuclei and more structural integrity.

### 2.5. EC-Exo Increases rCBF in MCAO/R Mice

**Figure 4:**
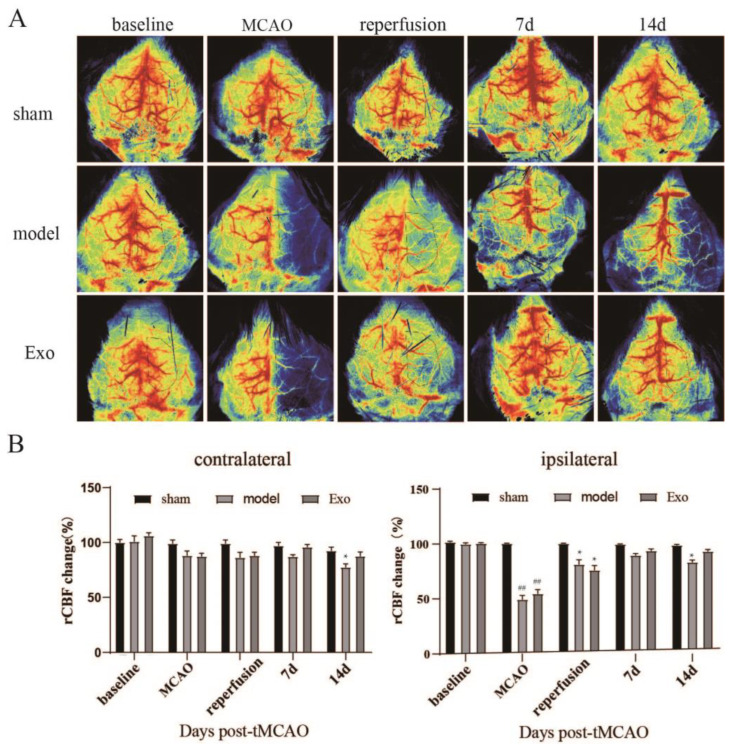
EC-Exo promotes recovery of cerebral blood flow after stroke. (**A**) before (baseline), 5 min during MCAO, reperfusion, 7 and 14 days after MCAO (black dashed lines indicate areas of concern for quantifying blood perfusion in MCA region; pseudocolor scales show high perfusion in red and low perfusion in blue). (**B**) Statistical results of rCBF changes expressed as a percentage of preischemic baseline in CL and IL hemispheres. The data are expressed as mean ± standard deviation (n = 10), The data were analyzed by one-way ANOVA followed by multiple comparisons using Tukey’s post hoc test. ## *p* < 0.01 vs. sham; * *p* < 0.05 vs. model. EC-Exo: exosomes derived from brain endothelial cells; MCAO: middle cerebral artery occlusion; MCA: middle cerebral artery; rCBF: regional cerebral blood flow; CL: contralateral; IL: ipsilateral.

To investigate the effect of EC-Exo on rCBF, we measured rCBF in mice using laser speckle imaging (LSI) before ischemia, immediately after ischemia, after reperfusion, and at 7 and 14 days. As illustrated in Figure 4, rCBF was significantly lower in the model group’s ischemic side than in the sham group (*p* < 0.05). Compared with the model group, rCBF in EC-Exo-treated mice was not significantly different at three days post-MCAO/R. The recovery of rCBF at seven days in the EC-Exo group was better than that in the model group, but there was an insignificant difference. The rCBF at 14 days in the EC-Exo group was significantly better than that in the model group (*p* < 0.05). Therefore, it was suggested that EC-Exo could increase rCBF in MCAO/R mice. The normal lateral rCBF in the model group was lower than in the EC-Exo group.

### 2.6. EC-Exo Improves Neurobehavioral Outcomes in MCAO/R Mice

**Figure 5:**
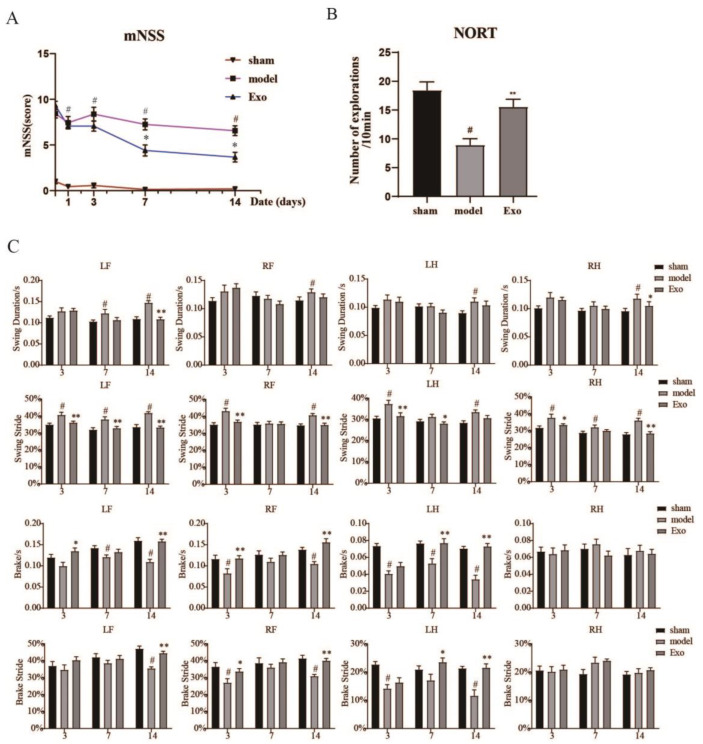
EC-Exo improves neurobehavioral recovery after stroke. (**A**) Mice mNSS scores immediately and 1, 3, 7, and 14 days after MCAO. The data are expressed as mean ± standard deviation (n = 16). The data were analyzed by Kruskal–Wallis analysis of variance. When statistical significance is obtained, Dunn’s test is applied. (**B**) Number of novelty explorations in mice after MCAO (n = 14). (**C**) Gait analysis results in mice 3, 7, and 14 days after MCAO (n = 14). The data are expressed as mean ± standard deviation, The data were analyzed by one-way ANOVA followed by multiple comparisons using Tukey’s post hoc test. # *p* < 0.05, vs. sham; * *p* < 0.05, ** *p* < 0.01 vs. model. EC-Exo: exosomes derived from brain endothelial cells; MCAO: middle cerebral artery occlusion; mNSS: modified neurological severity score; NORT: novel object recognition task; LF: left fore; RF: right fore; LH: left hind; RH: right hind.

mNSS scores were taken 2 h, 1, 3, 7, and 14 days after MCAO/R to investigate the effect of EC-Exo on neurobehavioral outcomes in mice. There were insignificant differences in mNSS between mice treated with EC-Exo and model mice after MCAO/R days 1 and 3. However, mice in the EC-Exo group performed significantly better than the model group on days 7 and 14, with significantly lower mNSS scores than the model group (*p* < 0.05) (Figure 5A). These results suggest that EC-Exo can attenuate neurological deficits in MCAO/R mice more than in the model group.

### 2.7. EC-Exo Treatment Improves Exploratory Behavioral Outcomes in MCAO/R Mice

The exploratory behavior of MCAO/R mice was assessed by the novel object recognition task (NORT) experiment. As demonstrated in Figure 5B, the mice in the model group were inactive and explored significantly less compared to the sham group (*p* < 0.05), while EC-Exo-treated mice performed significantly better than the model group (*p* < 0.05). This indicates that EC-Exo can improve the exploratory behavior of mice after MCAO/R.

### 2.8. EC-Exo Treatment Improves Gait Outcomes in MCAO/R Mice

We performed gait analysis on mice at 3, 7, and 14 days postoperatively to look for functional deficits caused by MCAO/R. Compared to the sham group, the mice revealed more pronounced abnormalities in limb movement on the contralateral side of the molding side due to brain injury. Due to a large amount of gait data, swing duration, % swing, braking duration, and % braking phase were selected here for a detailed description. The statistical results are displayed in Figure 5C, where the changes in the four limbs differed for the same parameters, most significantly in the left forelimb. EC-Exo group mice recovered 14 days after MCAO/R, while the model remained significantly impaired at the same time points for these spatial parameters, indicating that EC-Exo treatment induced faster gait recovery in MCAO/R mice.

### 2.9. EC-Exo Promotes Expression of Synaptic Plasticity Proteins in MCAO/R Mouse

**Figure 6:**
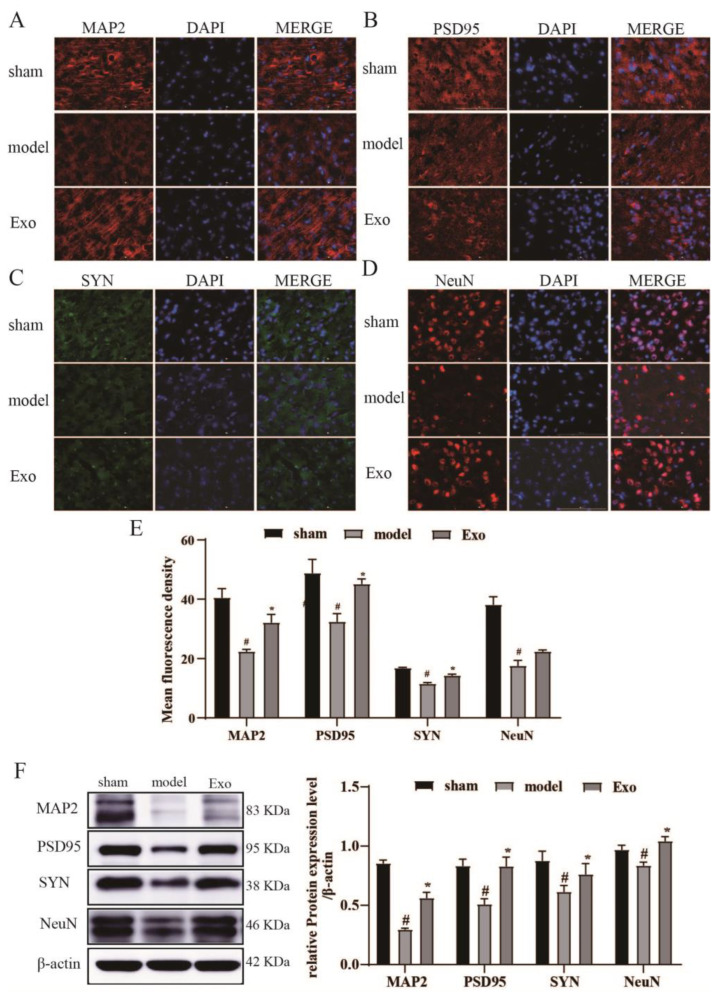
EC-Exo promotes synaptic remodeling after stroke. Immunofluorescence staining of (**A**), MAP2 (**B**), PSD95 (**C**), SYN, and (**D**) NeuN in frozen sections of mouse MCAO/R 14 days after brain freezing. Magnification ×400, Scale bar = 100 μm. (**E**) Mean fluorescence density statistics of MAP2, PSD95, SYN, and NeuN immunofluorescence staining in mouse MCAO/R brain frozen sections 14 days after MCAO/R. (**F**) Statistical results of MAP2, PSD95, SYN, NeuN protein expression, and protein expression in the cerebral cortex of mice 14 days after MCAO/R. The data are expressed as mean ± standard deviation (n = 4). The data were analyzed by one-way ANOVA followed by multiple comparisons using Tukey’s post hoc test. # *p* < 0.05, vs. sham; * *p* < 0.05, vs. model. EC-Exo: exosomes derived from brain endothelial cells; MCAO/R: middle cerebral artery occlusion/reperfusion.

To further investigate the effect of EC-Exo on synaptic remodeling in vivo, we examined changes in the expression of the neuronal cytoskeleton (MAP-2), presynaptic (synaptophysin, SYN), postsynaptic (PSD95), and other regulatory proteins of synaptic plasticity (NeuN) in brain tissue by IF staining and Western blotting (Figure 6). The results demonstrated that MAP2, PSD95, SYN, and NeuN expressions were significantly reduced in the model group compared to the sham group (*p* < 0.05) and significantly increased after the EC-Exo intervention (*p* < 0.05). No fluorescent signal was present in the negative control group. Statistical analysis of their fluorescence density, as displayed in Figure 6E, demonstrated that compared to the sham group, the model group had significantly lower protein expression levels of MAP2, PSD95, and SYN (*p* < 0.05), and those of the EC-Exo group were elevated. Western blotting (Figure 6F) was consistent with the IF staining results.

### 2.10. EC-Exo Inhibits Apoptosis in the Brain of MCAO/R Mice

**Figure 7:**
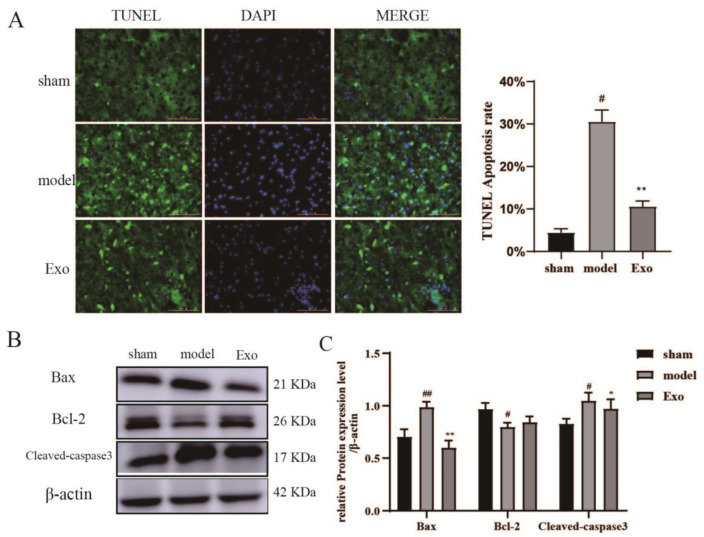
EC-Exo attenuate neuronal apoptosis after stroke. (**A**) Statistical results of apoptosis and number of apoptotic cells detected by TUNEL immunofluorescence in frozen sections of mouse brain 14 days after MCAO. (**B**,**C**) Statistical results of apoptosis-related protein expression in mouse cerebral cortex after 14 days of MCAO. The data are expressed as mean ± standard deviation (n = 4). The data were analyzed by one-way ANOVA followed by multiple comparisons using Tukey’s post hoc test. # *p* < 0.05, ## *p* < 0.01, vs. sham; * *p* < 0.05, ** *p* < 0.01 vs. model. Magnification ×200, scale bar = 100 μm. EC-Exo: exosomes derived from brain endothelial cells; MCAO: middle cerebral artery occlusion.

Next, we examined apoptosis in brain tissue by TUNEL staining and Western blotting to identify apoptosis-positive cells and associated protein levels. The number of TUNEL-positive cells was significantly higher in the model group (*p* < 0.05) and significantly lower in the EC-Exo treatment compared to the sham group (*p* < 0.05) (Figure 7A). No fluorescent signal was present in the negative control group. Furthermore, the expression of cleaved caspase-3 and Bax were significantly decreased after EC-Exo treatment, accompanied by an increase in Bcl-2 expression compared to the model group (*p* < 0.05) (Figure 7B,C). These results suggest that EC-Exo promotes synaptophysin expression and inhibits apoptosis in the brain of MCAO/R mice.

## 3. Discussion

Cerebral infarction is a common cerebrovascular disease characterized by neurological dysfunction caused by neuronal damage, which can be clinically improved by neuroprotective drugs [18]. Exosomes are cell-secreted nanoscale vesicles that can cross the BBB and have good biocompatibility and low immunogenicity [19]. Studies have demonstrated that exosome-mediated intercellular communication of multicellular origin plays a beneficial role in the pathophysiology of cerebral ischemia and has potential therapeutic value. Furthermore, as a cellular vesicle capable of crossing the BBB, exosomes have great promise in the endogenous drug-delivery system of the BBB in cerebral ischemia.

The successful isolation and purification of exosomes is an essential basis for the study of the physiopathological mechanisms of exosomes. Although there is no consensus on the gold standard for exosome isolation, most members of the International Society for Extracellular Vesicles (about 81%) choose to isolate exosomes by ultracentrifugation, independent of the biological sample selected [20]. In its guidelines, the International Society for Extracellular Vesicles (ISEV) gives a combined identification of exosomes by three experiments: particle size analysis, transmission electron microscopy, and marker proteins [21,22], which confirm the presence, number, and integrity of exosomes by cross-corroboration, confirming that the investigator has successfully isolated and purified exosomes and that subsequent findings based on this successful isolation and purification method can only be considered exosome-related studies. Therefore, we chose ultracentrifugation to isolate exosomes. The extracted exosomes were identified by transmission electron microscopy, particle size analysis, and Western blot of exosomal marker proteins CD9, CD63, and TSG101, to demonstrate that the exosomes obtained by this method conformed to the criteria.

Recent studies have displayed that EC-Exo plays a vital role in cellular communication. EC-Exo directly protects nerve cells from I/R injury by promoting neuronal growth, migration, and invasion [16]. EC-Exo markedly improved cognitive and neurological function in a type 2 diabetic mouse model, increasing arterial diameter, vascular density, and axonal density [17]. Despite some in vitro studies, the more positive effects of EC-Exo transplantation have not been fully elucidated, and more detailed experimental studies are needed. Therefore, the current study shows that EC-Exo has more positive effects on synaptic plasticity and other aspects.

Motor function is impaired after a stroke and affects the patient’s work and life. EC-Exo can significantly improve motor coordination in MCAO mice. Clinically, drugs that increase CBF at the ischemic site, enhance vascular compensatory capacity, and restore neurological function are hot research topics in treating ischemic stroke [23]. In this study, EC-Exo significantly reduced neurological function scores, increased cerebral blood flow to the ischemic side, and improved brain tissue structure in MCAO/R mice. Furthermore, our study found some effects on cerebral blood flow and neurobehavior in mice in the shorter postoperative period due to the damage caused by the surgery. The main effects were: some weight loss in the sham-operated mice, reduced cerebral blood flow to the normal side of the brain in all groups, and an insignificant difference in behavior between the immediate postoperative period and three days postoperatively.

MAP-2, which participates in microtubule assembly and growth, is explicitly found in dendritic branches of neurons and can be used as a marker of neuronal dendrites [24]. On the other hand, NeuN is a marker of mature neuronal cells and is reduced in MCAO/R rats as an alteration of neuron-specific genes [25]. This study demonstrated that MAP2 protein expression and NeuN-positive cells in brain tissue on the ischemic side were significantly increased after exosome treatment in cerebral ischemia.

Focal postinfarction damage to brain tissue is accompanied by changes in synaptic structure and function, including abnormal expression of synaptic plasticity proteins and altered synaptic density. It leads to neurological dysfunction in the body. The plasticity of synaptic structures is closely related to nerve growth and repair [26]. SYN expression indirectly reflects synaptic density and is closely related to neuroplasticity [27]. PSD-95 is a protein that acts as a marker for changes in synaptic functional activity [28] and is linked to synaptic plasticity and learned cognitive function in brain tissue. PSD95 and SYN protein expression is significantly reduced in brain tissue during ischemic injury [29]. The present study revealed that PSD-95 and SYN protein expression in brain tissue on the ischemic side was significantly increased after exosome intervention in cerebral ischemia. Furthermore, it was demonstrated that EC-Exo improved synaptic plasticity and learned cognitive function in cerebral ischemic mice.

Apoptosis is one of the modes of programmed cell death that is strictly controlled by multiple genes and is mainly regulated by the Bcl-2 family of proteins, which upon initiation, further activates the downstream molecule Caspase-3, which subsequently promotes apoptosis [30]. Caspase-3 is the most critical effector protease and a marker enzyme for the occurrence of apoptosis. It has been suggested that increased expression of Bcl-2 in the brain reduces the size of cerebral infarct lesions and has a protective effect on neurons. At the same time, Caspase-3, a downstream regulatory protein of Bcl-2, is effectively inhibited in its activity when Bcl-2 is overexpressed, thereby aborting the development of apoptosis [31]. In this study, EC-Exo significantly reduced apoptosis in primary neurons in vitro. Furthermore, EC-Exo significantly reduced Bcl-2 and cleaved Caspase-3 expression in brain tissue of MCAO/R mice and increased Bax expression in brain tissue compared with the model group. The results showed that EC-Exo could improve apoptosis in cerebral ischemia mice.

## 4. Materials and Methods

### 4.1. Cell Culture

Endothelial cell bEnd.3 (ATCC Cat# CRL-2299) was isolated from the brain tissue of mice with endothelioma. Its applications include neuroscience research. Cells were cultured in Dulbecco’s modified Eagle medium (DMEM, #C11995500BT, Gibco, NY, USA) with 10% fetal bovine serum (FBS, #10099-141C, Gibco, NY, USA. Centrifugation at 100,000× *g*, 4 °C for 16 h was performed to remove serum exosomes) and 1% penicillin–streptomycin solution (PS, #15140-122, Gibco, NY, USA) in a humidified environment with 5% CO_2_ at 37 °C.

### 4.2. Exosome Extraction and Characterization

Exosomes were purified from the cell culture supernatant as described previously [32]. Briefly, after replacement with serum-free medium, the supernatant of bEnd.3 cell medium was collected, centrifuged at 300× *g* for 10 min at 4 °C, and then centrifuged for a further 20 min at 2000× *g* to remove dead cells. Next, they were transferred to a new centrifuge tube after 10,000× *g* and centrifuged at 4 °C for 30 min to remove cellular debris. After passing the cell supernatant through a 0.22 μm filter membrane (Millipore, MA, USA), they were transferred to an ultracentrifuge tube and centrifuged at 100,000× *g* for 70 min at 4 °C. They were then poured off the supernatant, and the precipitate was resuspended in PBS and centrifuged at 100,000× *g* for 70 min at 4 °C, and the precipitate was resuspended in 200 μL of PBS. The exosomes were stored at −80 °C to prevent degradation.

The extracted exosomes were resuspended in PBS, dropped onto a 2 nm pore size carrier copper mesh, and left to stand for 3 min at room temperature. They were then blotted dry with filter paper from the mesh side, negatively stained with 2.5% phosphotungstic acid solution (#P1126, Solarbio, Beijing, China) for 10 min at room temperature, and blotted dry with filter paper. Finally, they were dried at room temperature and photographed for electron microscopy (Hitachi, Tokyo, Japan).

The Malvern Nano ZS (Malvern Instruments, Malvern, UK) was used to assess the number and size of exosomes. The exosomes obtained by ultracentrifugation were diluted with PBS and subjected to particle size analysis. The concentrations of proteins were measured using the BCA protein assay kit (#23227, Thermo Scientific Pierce, Rockford, IL, USA).

### 4.3. Primary Mouse Neuronal Cultures

Primary neurons were prepared from the cerebral cortex of neonatal C57BL/6J mice. Briefly, the cerebral membranes were separated and stripped in ice-cold DMEM/F12 (#C11330500BT, Gibco, NY, USA). Then, the cortex was isolated, digested in 0.25% trypsin-EDTA (#25200-072, Gibco, NY, USA) for 10 min, and the dissociated cortical cells were spread in 24-well and 96-well plates coated with poly-D-lysine (#P4707, Sigma-Aldrich, MO, USA). Four hours after inoculation, the medium was replaced with a basal neural medium. Cells were cultured at 37 °C in a humidified incubator containing 5% CO_2_. The cell culture medium was changed in half volumes every other day.

### 4.4. CM-DiI Labeling of Exosome and Cell Uptake of Exosomes

Exosomes were labeled with the orange fluorescent membrane dye CM-Dil (C7000, Thermo Fisher, MA, USA) [33]. Briefly, the configured dye working solution (1 μM) and the exosomes (5000 μg/mL) were mixed by vortexing at a 1:1 ratio for 1 min and were incubated at 37 °C for 30 min. To remove excess dye, 10 mL of PBS was added to the exosome dye mixture, and the supernatant was centrifuged at 100,000× *g* for 2 h at 4 °C. In the final centrifugation step, the stained exosome precipitate was suspended in PBS (0.5 mL). Finally, the solution was filtered through a 0.2 μm membrane filter to remove dye aggregates.

Exosomes labeled with CM-Dil were added to the medium of primary neuronal cells and incubated at 37 °C for 12 h, protected from light. After fixation, cells were stained with DAPI (#C0060, Solarbio, China) (for staining nuclei) and MAP2 (#17490-1-AP, Proteintech, Chicago, IL, USA) (for staining neuronal cells). Finally, imaging analysis was performed using the IN-Cell Analyzer 2500HS system.

### 4.5. OGD/R Model and Exosome Treatment

The primary neuronal medium was changed to serum- and glucose-free DMEM (#11966-025, Gibco, NY, USA) on day 8 of in vitro culture to establish the OGD model. After 2 h, the cells were removed from the anoxic chamber (Thermo Fisher, MA, USA), filled with 5% CO_2_ and 95% N_2_, and treated with exosomes. In this experiment, a similar dosing regimen [34] was used and optimized based on it.

### 4.6. Cell Viability Assays

The cells were incubated for 24 h, and the supernatant was discarded. Next, 100 μL of CCK-8 working solution (#CK04, DOJINDO, KM, Japan, final concentration 10%) was added to each well and incubated for 120 min at 37 °C. The absorbance value of each well was measured at 450 nm by an enzyme marker and correlated positively with cell viability.

### 4.7. MCAO/R Model and Exosome Treatment

In total, 48 male C57BL/6J mice, weighing 23 ± 2 g, were purchased from Beijing Viton Lever Laboratory Animal Technology Co Ltd. (Beijing, China). The mice were placed at 25 °C under a 12 h light–dark cycle and fed with standard food pellets and drinking water. All animal experiments were conducted in accordance with the relevant regulations and guidelines of the National Institute of Health, Guide for the Care and Use of Laboratory Animals, and with the permission of Tianjin University of Traditional Chinese Medicine’s Animal Care and Use Committee (Tianjin, China; Permit No. TCM-LAEC2021246, 14 November 2021). The relevant observers were blinded to the grouping of all experiments and the assessment of all experiments. According to the MCAO model described previously [35], mice were anesthetized with isoflurane (#R510-22, Rivard, Shenzhen, China) mixed in 70% N_2_ and 30% O_2_. The right common carotid artery was isolated, a 0.20 ± 0.01 mm nylon silicone coated monofilament was inserted (A5-1620, Sinon, Beijing, China), and the filament was gently placed pushed into the MCA. The filament was removed after 90 min of occlusion to allow for reperfusion. Mice in the sham-operated group underwent the same procedure, except for the occlusion process. During the procedure, mice were kept on a thermostatic heating pad at 37 °C, and their body temperature was monitored. At 24 h after reperfusion, the neurobehavioral outcome of the mice was scored using the Zea-Longa score. Neurological results were scored on a 5-point scale [36]. Mice with a score of 1–3 were considered successful for MCAO/R, while mice with a score of 0 or 4 were excluded from the study.

The mice were treated 2 h after reperfusion, similar to when most stroke patients receive treatment (144 min) [37]. Exosomes (60 μg of total protein diluted in 100 μL PBS, n = 16) or PBS alone (100 μL, n = 16) were injected into the tail vein of mice and dosed for seven consecutive days. The dose was chosen based on previous laboratory studies. The dose was chosen based on previous studies [16,34,38] and pre-experiments in which rats were given 100 μg exosomes intravenously and scaled down according to the body weight of the mice, and our pre-experimental results showed that 60 μg dose was more effective of the mice.

### 4.8. Fluorescently Labeled Exosomes and In Vivo Tracking Studies

Exosomes were labeled with the NIR membrane dye DIR (#UR21017, Umibio, Shanghai, China). The stained exosome precipitate was suspended in PBS. The solution was filtered through a 0.2 μm membrane filter to remove dye aggregates.

The small animal in vivo fluorescence imaging module was evaluated using Optical Imaging System (IVIS^®^ LuminaK Series, PerkinElmer, MA, USA). DIR-labeled exosomes (60 μg/only) were injected into mice through the tail vein. Exosome in vivo tracing was observed 0.5, 3, 6, 12, 24, and 48 h after injection.

### 4.9. Measurement of rCBF

RFLS III, a full-field laser perfusion imager (RWD Life Sciences, Shenzhen, China), was used for mouse brain laser scatter imaging. Briefly, after anesthesia, an incision was made in the middle of the mouse scalp to expose the skull, and laser speckle imaging was performed by the imager located directly above the skull. In addition, we measured cerebral blood flow in mice before and after ischemia, at reperfusion, and after 7 and 14 days.

### 4.10. Examination of Neurological Functions

A modified neurological severity score (mNSS) is also used to evaluate neurological function. The mNSS comprehensively assessed motor balance and sensory functions. Neurological function was graded from 0–18 (1–6 being mild, 7–12 being moderate, and 13–18 being severe). All assessments were performed by three individuals who did not know the experimental design.

### 4.11. Gait Analysis

The DigiGait System (mouse-specific) was used for gait capture and analysis. First, mice were acclimatized three days in advance. Before recording the video, mice were allowed to acclimatize in a running room for 3 min and then run at 10 cm/s to get used to the belt movement. Next, the viewing frame, illuminance, and contrast were adjusted to ensure that the mice’s paws could be seen clearly in the captured images. The belt speed was then set at 15 cm/s, and the gait was recorded. The video was recorded until at least three 3 s periods of uninterrupted running were obtained (during the run, the rat did not stop, jump, or place its paws on the room’s walls). Once the footage was acquired, it was postprocessed and analyzed using the software provided with the DigiGait imaging system.

### 4.12. NORT

Behavioral recordings lasting 10 min were performed on day 14 after MCAO/R to assess the mice’s object recognition and memory abilities. Before the test, mice were allowed to move freely within the experimental apparatus (without objects) for 10 min to acclimatize to their environment, excluding environmental factors. On the second day, two identical objects were placed in the apparatus, and the mice were placed with their backs facing the objects from an equal distance from the apparatus for 10 min. The camera equipment and software were used to record the number of times the mouse explored each object (touching the object with its mouth or nose) within 10 min. At the end of each mouse, the training field and objects were wiped with a 75% ethanol solution. All behavioral experiments required quiet surroundings and were simultaneously conducted by the same staff.

### 4.13. Crystalline Violet Staining

Crystalline violet staining was used to observe the area of brain tissue injury. Coronal sections (4 μm) were dewaxed, dehydrated, stained using crystalline violet solution (#G1061, Solebro, Beijing, China), and observed and photographed using a pathology scanner (Pannoramic MIDI, BP, Hungary).

### 4.14. HE

Hematoxylin and eosin (HE) staining was performed to examine the histopathology of brain tissue after injury. Coronal sections (4 μm) were deparaffined, dehydrated, and stained using a modified HE kit (#G1120, Solebro, Beijing, China). They were then observed and photographed using a pathology sweeper (Pannoramic MIDI, BP, Hungary).

### 4.15. TUNEL Assay

Cells and frozen brain tissue sections were stained according to the instructions from the One Step TUNEL Apoptosis Assay Kit (#C1088, Beyotime, Shanghai, China). PBS was used as a negative control instead of antibody. Five randomly selected fields per well/brain slice were photographed at 200 × field of view. Nuclei with identically labeled green fluorescence were selected using ImageJ software as a uniform criterion for judging all positive cells, and DAPI blue nuclei were chosen as total cells. Five photographs were randomly selected for each sample to calculate the proportion of TUNEL-positive cells.

### 4.16. Western Blot Analysis

SDS-PAGE was used to separate the proteins from exosomes and animals. Electrophoretic transfer was performed to polyvinylidene difluoride (PVDF) membranes (Millipore, MA, USA) with TSG 101 (1:1000 dilution; #ab125011, Abcam, MA, USA), CD9 (1:1000 dilution; #ab92726, Abcam, MA, USA), CD63 (1:1000 dilution; #ab68418, Abcam, MA, USA), MAP2 (1:5000 dilution, #17490-1-AP, Proteintech, Chicago, IL, USA), SYN (1:1000 dilution, #ab8049, Abcam, MA, USA), PSD95 (1:1000 dilution; #2507S, CST, MA, USA), NeuN (1:5000 dilution; #ab177487, Abcam, MA, USA), Bax (1:2000 dilution; #BS6420, Bioworld, MN, USA), bcl2 (1:500 dilution; #BS1511, Bioworld, MN, USA), caspase3 (1:500 dilution; #BS7073, Bioworld, MN, USA) overnight at 4 °C. Incubation with horseradish peroxidase-conjugated anti-mouse or anti-rabbit antibodies (#ZB2305 or #ZB2301) (1:10,000 dilution; Zhongshan Jinqiao, Beijing, China). Finally, immunoblot assays were performed using ECL chemiluminescent solution (#WBKlS0100, Millipore, MA, USA). Western blot quantitative analysis was performed using Image-Pro Plus 6.0.

### 4.17. Immunostaining

Primary neuronal cells and frozen sections of brain tissue were fixed at room temperature in 4% paraformaldehyde and washed lightly with PBS thrice. MAP2 (1:200 dilution, #17490-1-AP, Proteintech, Chicago, IL, USA), SYN (1:250 dilution; #ab8049, Abcam, MA, USA), PSD95 (1:200 dilution; #2507S, CST, MA, USA), and NeuN (1:500 dilution; #ab177487, Abcam, MA, USA) antibodies were added and incubated overnight at 4 °C. PBS was used as a negative control instead of antibody. On the second day, the cells and slices were washed with PBS, protected from light by adding IgG-FITC (1:1000) fluorescent secondary antibody, and incubated for 60 min at 37 °C. The slices were washed with PBS, nucleated by adding DAPI stain, blocked with an antifluorescence quencher, and observed under an inverted fluorescence microscope. Non-overlapping areas of the ischemic cortical region were randomly selected for imaging. ImageJ software was used to analyze fluorescence intensity and positive cell counts.

### 4.18. Statistical Analysis

All experimental data were analyzed using SPSS 23.0 software and GraphPad Prism 8.0. All sample data conformed to Levene’s analysis of equal variance (*p* *<* 0.05) and the Shapiro–Wilk test for normal distribution (*p* < 0.05). The data were analyzed by one-way ANOVA followed by multiple comparisons using Tukey’s post hoc test. In addition, nonparametric data using Kruskal–Wallis analysis of variance and Dunn’s test were used when statistically significant. *p* < 0.05 was considered statistically significant.

## 5. Conclusions

In summary, exosomes were obtained and identified using differential centrifugation. To demonstrate the therapeutic effect of EC-Exo on ischemic stroke, we first observed by in vitro experiments that EC-Exo increased primary neuronal survival, increased synaptic length, and reduced neuronal apoptosis after OGD/R. Based on the in vitro studies, we further demonstrated in vivo the therapeutic effects of EC-Exo in mice with ischemic stroke. In vivo experiments demonstrated that EC-Exo increased cerebral blood flow, improved neurobehavior and motor behavior, reduced ischemia-induced apoptosis, and promoted synaptic remodeling in MCAO/R mice. These findings suggest that EC-Exo mediates the therapeutic effects of neurorepair in MCAO/R mice and that its beneficial effects on ischemic stroke treatment are related to the effects of exosomes in increasing neuronal survival antiapoptosis and promoting synaptic remodeling. These findings support the potential clinical benefit of EC-Exo treatment for ischemic stroke. Although cellular and animal studies have been performed, the mechanisms by which EC-Exo transplantation has a positive effect have not been fully elucidated and the current data do not yet describe the specific molecules in exosomes that are involved in this process. Further studies are needed in the future to demonstrate the specific molecular mechanisms by which EC-Exo exerts its neuroprosthetic effects.

## Data Availability

Data are contained within the article.
